# Maintained Improvement of Neurocognitive Function in Major Depressive Disorders 6 Months after ECT

**DOI:** 10.3389/fpsyt.2016.00200

**Published:** 2016-12-19

**Authors:** Christine Mohn, Bjørn Rishovd Rund

**Affiliations:** ^1^Research Department, Vestre Viken Hospital Trust, Drammen, Norway; ^2^Department of Psychology, University of Oslo, Oslo, Norway

**Keywords:** cognition, depression, ECT, MCCB, memory, neuropsychology

## Abstract

Both impaired and improved cognitive function after electroconvulsive therapy (ECT) in major depressive disorder (MDD) patients may occur. We have previously found improved cognitive function 6 weeks after ECT in this group. The aim of this study was to report 6-month follow-up results from the same prospective project monitoring cognitive effects of ECT. Thirty-one patients with MDD were assessed with the MATRICS Consensus Cognitive Battery (MCCB), the Everyday Memory Questionnaire (EMQ), and the Montgomery–Åsberg Depression Rating Scale prior to, 6 weeks, and 6 months after ECT. Compared to baseline, the speed of processing, attention/vigilance, and reasoning/problem solving test results were significantly improved. The depression score was significantly reduced. There were no changes in subjective memory complaint. There was no significant relationship between the EMQ and the MCCB subtests, but a significant correlation between current depression level and the EMQ. Six months after ECT the cognitive improvement reported at 6-week follow-up was maintained and extended. The corresponding decrease in depressive symptoms and stability in subjectively reported memory complaints suggest that the antidepressant effects of ECT do not occur at the expense of cognitive function.

## Introduction

Electroconvulsive therapy (ECT) is a potent method for inducing symptom relief in treatment-resistant depression. However, fear of cognitive side effects, such as memory loss and confusion, is often cited as the main reason for not consenting to this type of treatment (1).

The literature on cognitive function after ECT for major depressive disorders (MDDs) is inconclusive, with reports of both impaired (2) and improved (3) cognitive capacity during the first weeks after treatment. Limitations of previous research are lack of longitudinal studies beyond the first few months after ECT and the assessment of few cognitive functions. The Sackeim et al. (2) study presents comprehensive cognitive function data from a relatively long time period after ECT (6 months) and reports deficiencies in speed of processing and autobiographic memory at this time point, whereas the other cognitive functions assessed were returned to normal. However, their participant group was heterogeneous in that some of the patients received square wave, brief pulse stimulation, while others were treated with sine wave ECT. Sine wave stimulation is no longer recommended, as it is related to stronger cognitive side effects than square pulse methods (4). Thus, there is a need for longitudinal studies of the effects of square wave stimulation. Currently, we follow depression patients for 2 years after square wave, brief pulse ECT, employing a comprehensive cognitive test battery, the MATRICS Consensus Cognitive Battery (MCCB) (5).

The MCCB was developed for neurocognitive assessment in schizophrenia spectrum disorders but may also be of utility in bipolar disorder (6–8) and MDD patients (9). The Norwegian version of the battery is currently used for monitoring cognitive effects of ECT in bipolar disorder (10) and MDD (11). A particular advantage of the MCCB is that it consists of tests assumed to withstand test–retest effects and thus permits repeated testing within relatively short time spans without biasing the results due to learning effects.

Previously, we have reported statistically significant improvements in cognitive function, i.e., speed of processing, attention/vigilance, and visual learning, 6 weeks after ECT as compared to baseline (11). Moreover, there was no significant change in subjective cognitive complaints from baseline, although the post ECT self-report of cognitive complaints was correlated with residual depression symptoms (11). This previous study gives cause for optimism regarding cognitive side effects of ECT. However, the first few weeks after treatment is usually an unstable period for many patients, with fluctuations in side effects, discharge from hospital, and social reorientation. The aim of the present paper is to assess cognitive function of the participants of the 6-week study at a time when the clinical picture is more stable, i.e., 6 months after ECT.

## Materials and Methods

### Participants

We assessed 31 Caucasian participants with an MDD episode recruited from the ECT clinical sections at Vestre Viken Hospital Trust and Vestfold Hospital Trust in South-Eastern Norway from March 2011 to November 2014.

At both of these time points, 31 patients participated. However, two of the patients from the 6-week follow-up test did not participate at 6 months due to relapse and excessive fatigue. Moreover, at the current time point, two of the patients who had been included at baseline but not participated at the previous assessment had recovered and were able to be tested. Therefore, the demographic data presented in Table 1 are slightly different from those described previously (11).

**Table 1:** **Demographic characteristics of the participants (*N* = 31)**.

Age (years)	46.0 (SD 10.4)
Gender	*n* = 11 (35.5%) men
	*n* = 20 (64.5%) women
Education	
Elementary school	*n* = 8 (25.8%)
High school	*n* = 13 (41.9%)
BA/BA+	*n* = 10 (32.3%)
Years since first onset of depression	20.8 (SD 10.5, range 5–40)

*Age and years since onset in mean*.

*BA, Bachelor degree*.

Inclusion criteria were age above 18 and below 70 years, capacity for giving informed consent to both ECT and participation in this project, ability to understand spoken and written Norwegian, and a diagnosis of a treatment-resistant major depressive episode. The diagnosis of “treatment-resistant depression” was made by the clinicians based on previous lack of response to antidepressant medication in combination with psychotherapy. Exclusion criteria were ongoing alcohol or drug abuse, ongoing neurological illness, and ECT within the last 2 years.

### Clinical Assessment

The diagnosis of a major depressive episode (F 32.1, F 32.2, F 32.3) was established by clinical interviews by hospital staff according to the ICD-10 criteria (12). Several different clinicians were involved in the diagnostic process. The patients were severely ill, and the decision to commence ECT was sometimes made so rapidly that the diagnostic process could not be undertaken by one and the same clinician. We did, however, rely on comprehensive information from the patients’ files to support the diagnosis made according to the ICD-10 system.

Twenty-four of the patients were diagnosed with recurrent unipolar depression (F 33) and seven with bipolar disorder type II (F 31) (12). Six had experienced psychotic symptoms during depressive episodes, 10 had moderate anxiety symptoms, and 4 partially fulfilled the criteria for a personality disorder (emotionally unstable personality disorder, F 60.3, and anxious personality disorder, F 60.6). Severity of depression was assessed with the Montgomery–Åsberg Depression Rating Scale (MADRS) (13), at the start of the neuropsychological assessment session.

Four of the patients had been treated with 1–2 series of ECT more than 2 years previously. All patients had discontinued their psychotropic medication 1–7 days before baseline testing. Medication for anxiety and/or insomnia had been permitted the evening before baseline testing. Two patients did not use any regular medication at baseline, and five were medicine-free at 6 months after ECT. Calculated daily doses (CDD) of medication were based on the international daily doses of medication technical measurement system (14) (Table 2). A CDD is the outcome of the prescribed daily dose divided by the average recommended daily dose of medication.

**Table 2:** **Medication (CDD) before and 6 months after ECT**.

	Pre ECT (*N* = 29)	Post ECT (*N* = 26)
Antidepressants	2.5	1.5
Antipsychotics	1.1	0.8
Lithium	0.8	0.8
Anticonvulsants	0.6	0.6

*CDD, calculated dose of medication based on the prescribed dosage divided by the average recommended daily dosage*.

### Electroconvulsive Therapy

No uniform ECT procedure was followed, and each treatment procedure was tailored to the individual patient. All patients received square wave, brief pulse (0.5 ms) stimulation from a Thymatron system machine two (*n* = 3) or three (*n* = 28) times a week. The stimulation dose was age-based, i.e., the electrical current delivered was based on the patient’s age as percentage of 504 mC. With unilateral electrode placement, the stimulation was 4–6 times above seizure threshold and 2.25 times above seizure threshold with bilateral placement. Mean number of applications per ECT series was 11.9 (SD 4.1, range 6–23). Right unilateral electrode placement was used in 22 cases, bifrontal placement in 1 case, and mixed placement (switching from right unilateral to bifrontal in mid-series) in 8 cases. Anesthetic agents were alfentanil, propofol, or thiopental. Succinylcholine was used as a muscle relaxant. These pharmacological agents were administered in dosages according to the physical characteristics of each participant.

After this first series and between the 6-week follow-up assessment, 6 patients received maintenance treatment with a mean 11.3 of applications (SD 6.0, range 5–18). Among these, four had right unilateral, one bifrontal, and one mixed electrode placement.

Between the 6-week and 6-month follow-up tests, 1 patient received a new series of ECT (9 applications) and maintenance treatment (13 applications) with right unilateral electrode placement.

The participants were cognitively assessed 1–3 days before the start of ECT, 6 weeks, and 6 months after completion of ECT. For the patients who received maintenance treatment, the follow-up cognitive assessments were performed 6 weeks or 6 months after the final application of ECT.

All participants signed an informed consent form before both testing sessions. These consents were given in addition to their consenting to ECT treatment, which was obtained by the clinical departments. The study was conducted according to the Helsinki Declaration and was approved by the Regional Committee for Research Ethics for Health Region South-East (REK Sør-Øst).

### Neuropsychological Assessment

The cognitive assessment was carried out by a clinical psychologist with extensive neuropsychological training (CM). The participants were tested at their respective clinical wards at baseline and at their respective outpatient clinic or at home at follow-up.

The MCCB covers 7 cognitive domains using 10 subtests (5, 15):

Speed of Processing, consisting of the subtests Trail Making Test A (TMT-A) (16), Symbol Coding [Brief Assessment of Cognition in Schizophrenia; (17)], and Fluency [Category Fluency; (18)],
Attention/Vigilance, assessed by The Continuous Performance Test-Identical Pairs (CPT-IP) (19),
Working Memory, consisting of the subtests Spatial Span [The Wechsler Memory Scale; (20)] and Letter Number Span [The University of Maryland Letter Number Span test; (21)],
Verbal Learning, assessed by the revised Hopkins Verbal Learning Test [immediate recall; (22)],
Visual Learning, measured by the revised Brief Visuospatial Memory Test (23),
Reasoning/Problem Solving, assessed by the Mazes test [Neuropsychological Assessment Battery; (24)], and
Social Cognition, measured by the Managing Emotions part of the Mayer–Salovey–Caruso Emotional Intelligence Test (MSCEIT) (25).

For a detailed description of the tests and the Norwegian standardization process, see Mohn et al. (26). Although originally developed for schizophrenia spectrum disorder patients, we have previously demonstrated that the MCCB is capable of generating a separate neurocognitive profile in MDD patients compared to healthy controls (27).

The average time of completion of the MCCB is approximately 60 min. Due to excessive fatigue, 4 patients did not perform the MSCEIT and 10 did not perform the CPT-IP test.

Norwegian *T* scores have been published for the 20–59 years age group (26). As the current study includes participants above 60 years, the MCCB results are presented in raw scores.

After the completion of the MCCB, the patients filled in the Everyday Memory Questionnaire (EMQ) (28), assessing practical attention and memory functions in 28 items.

### Statistics

All statistical analyses were performed using IBM SPSS Statistics version 22. As we had three time points (baseline, 6 weeks, and 6 months) and random missing data (for MSCEIT and CPT-IP, see above), a linear mixed model approach was appropriate for analyzing differences across time. In all models, a score of the MADRS, EMQ, or a cognitive test was the dependent variable, and time the fixed factor.

The relationships between the EMQ and the MCCB scores were investigated with Pearson’s correlations.

## Results

There was a large, significant reduction in the MADRS score from baseline to 6 weeks and 6 months. The EMQ score did not significantly change across time. The scores of 5 of the 10 MCCB tests were significantly improved across time: TMT-A, Symbol Coding, Fluency, Mazes, and CPT-IP, assessing the domains speed of processing, reasoning/problem solving, and attention/vigilance, respectively. The cognitive functions assessed by the other five subtests were unaltered across time. At no point was there any cognitive deterioration compared to baseline (Table 3).

**Table 3:** **Depression levels, subjective cognitive function, and MATRICS Consensus Cognitive Battery test scores (raw scores) of the participants (*N* = 31)**.

	Baseline mean (SD)	6 weeks post ECT mean (SD)	6 months post ECT mean (SD)	*T* (sign.)
**Montgomery–Åsberg Depression Rating Scale**	33.8 (8.1)	15.6 (9.2)	17.7 (8.3)	−7.01***
**Everyday Memory Questionnaire**	104.0 (37.9)	107.9 (43.6)	98.5 (42.6)	−0.67
**Speed of processing**				
Trail Making Test A	48.7 (25.8)	39.8 (21.4)	37.0 (16.5)	−3.00**
Symbol coding	40.5 (13.0)	44.6 (12.0)	46.4 (12.9)	3.57***
Fluency	21.3 (8.5)	22.2 (6.4)	24.5 (7.0)	2.98**
**Working memory**				
Spatial Span (The Wechsler Memory Scale)	13.2 (3.0)	13.6 (3.2)	14.2 (3.3)	1.88
Letter Number Span Test	12.3 (3.9)	12.6 (3.8)	12.6 (3.4)	0.74
**Verbal learning**				
Revised Hopkins Verbal Learning Test	22.6 (6.0)	23.2 (6.2)	23.1 (6.3)	0.48
**Visual learning**				
Revised Brief Visuospatial Memory Test	21.4 (8.6)	23.5 (8.0)	23.7 (7.6)	1.74
**Reasoning/problem solving**				
Mazes	13.5 (8.1)	13.9 (7.9)	16.1 (7.7)	2.62*
**Social cognition (*n* = 27)**				
Mayer–Salovey–Caruso Emotional Intelligence Test	93.7 (8.0)	94.6 (9.5)	95.3 (8.4)	1.40
**Attention/vigilance (*n* = 21)**				
Continuous Performance Test-Identical Pairs	2.5 (0.5)	2.7 (0.5)	2.8 (0.5)	4.12***

*Results of the linear mixed model analyses*.

*P < 0.05, **P < 0.01, ***P < 0.001.

There was no significant association between the EMQ score and the MCCB test results (*P* > 0.05). The correlation between the MADRS and the EMQ scores was statistically significant (*r* = 0.49, *P* < 0.01).

## Discussion

### General Discussion

We have demonstrated that our previous findings of significantly improved neurocognitive function 6 weeks after ECT (11) are maintained at the 6-month follow-up point. The improvement of the subtests of the speed of processing (TMT-A, Symbol Coding, and Fluency) and attention/vigilance (CPT-IP) domain we reported at 6 weeks was sustained at 6 months. The other test results were stable across time, with no deterioration at any point. Moreover, the improvement at 6 months was extended to the reasoning/problem solving (Mazes) domain. The tests assessing these cognitive domains all rely on time, as high scores partly depend on fast task performance. Processing speed is among the functions most strongly affected by depression (27, 29), and clinical remission is likely to be accompanied by increased performance speed.

Improved neurocognitive function after ECT has been demonstrated by others 15 days (3), 1 month (30), and 3 months (31) after cessation of treatment. At 6 months after ECT, Sackeim et al. (2) reported cognitive improvement in most domains assessed, with the notable exception of processing speed, which was still compromised. However, the authors explain that finding as the likely result of the employment of sinus wave stimulation in a sizable minority of the participants.

It may be argued that our results are influenced by test–retest effects. However, one of the aims behind the MCCB test selection and standardization procedure was the development of a battery that allows repeated assessments at relatively short intervals, and alternate forms of the tests are used when appropriate (32). A recent study of schizophrenia patients demonstrated limited practice effects in MCCB test results during a 4-week follow-up period (33). Thus, with repeated assessments over longer intervals than 4 weeks, we have little reason to expect this type of bias.

There are several ways in which these results may be interpreted. First, it is possible that ECT boosts cognitive performance directly by inducing transient neuroplastic changes in the hippocampus and other cerebral regions involved in learning and memory (34). Alternatively, ECT may have indirect effects and lead to increased cognitive function *via* antidepressant properties. Impaired cognitive function is common in depressive disorders (27, 29, 35), and cognitive performance may naturally increase following symptom remission. A third possibility is the joint neuroplastic and antidepressant effect of ECT. To our knowledge, no study has elucidated this topic through a combination of brain imaging and behavioral measures, and we are unable to determine which of these hypotheses best explains our results.

Across time, there was no statistically significant change in subjective memory problems, as assessed by the EMQ. Of note, there was no significant correlation between the EMQ and objective neuropsychological performance at the current time point. Hence, the modest association between the EMQ and two MCCB tests we obtained at 6 weeks had disappeared (11), and the cognitive problems our participants may experience on a daily basis were not reflected in the test scores. This discrepancy may be explained by the depressed participants’ low confidence in their own cognitive capacity. Alternatively, the EMQ and the MCCB tap different aspects of cognitive function, the former providing an estimate of naturalistic function, and the latter assessing a few functions during a highly structured test situation with thorough instructions of behavior.

Relatedly, in our previous study of the same participants 6 weeks after ECT, we found a statistically significant positive association between the EMQ score and the MADRS score (11). We found the same relationship in the current study. This indicates that the depressive symptoms and the subjective experience of cognitive dysfunction may be two sides of the same coin (11, 36). A limitation of our study is that we did not have sufficient statistical power to analyze subjective and objective cognitive function in subgroups divided according to their posttreatment symptoms of depression, so the above suggestion remains somewhat speculative. Nevertheless, when monitoring cognitive effects of ECT, we recommend that both objective and subjective function is recorded and seen in relation to residual depression symptoms.

### Strengths and Limitations

Major strengths of this study are the employment of an internationally standardized, comprehensive cognitive test battery, a low drop-out-rate, a long follow-up period, and the cognitive assessment being performed at a stable clinical point.

The main limitation of our study is the relatively low *N*, rendering separate analyses based on gender, age, and diagnostic subgroups impossible. Women and elderly patients may display stronger cognitive side effects of ECT (2). Moreover, patients with symptoms of bipolar disorder or psychosis are generally more cognitively disadvantaged than MDD patients without such additional symptoms (37, 38), and it may be speculated that these patient groups exhibit different cognitive profiles after ECT. The fact that we were not able to control for these factors limits the generalizability of our results.

Relatedly, the low *N* may have generated type I errors. With more conservative statistics, some of our significant effects may not have occurred. Due to the exploratory nature of our study, however, we chose not to correct for multiple comparisons.

In addition, the ECT was not standardized, but tailored to each patient’s clinical characteristics. In our sample, both unilateral and bilateral electrode placement was used, and unilateral stimulation may generate less cognitive side effects than bilateral placement (2). A larger *N* would have enabled us to provide statistical control for the effects of electrode placement. Therefore, although ecologically valid, the present study may not satisfy the strictest methodological requirements.

A second limitation is our lack of control group comparisons. Analyses of cognitive function data from a group of MDD patients undergoing pharmacological and/or psychotherapeutic treatment would have enabled us to isolate the cognitive effects of ECT. However, a study comparing the MCCB test scores after ECT or pharmacological treatment of bipolar disorders (10) found no group differences in cognitive outcome. Still, a control group comparison would have put our findings on a more secure footing.

A third limitation is the fact that our participants were able to give their informed consent to ECT as well as to participation in this study. Our results may not generalize to those whose depression level is so high that they are unable to participate in research.

Finally, we did not assess autobiographical, episodic memory, a function that is often assumed to be negatively affected by ECT (1). The main reasons were the wish to spare a severely ill and fatigued group of participants another time consuming test and lack of psychometrically strong assessment tools of retrograde cognitive function in depressed individuals (39).

### Conclusion and Clinical Implications

This is the second report from our longitudinal study of the cognitive effects of ECT. Six months after treatment, cognitive improvement was maintained and expanded from the previous assessment at 6 weeks. There was no increase in subjective memory complaints after ECT. Despite methodological limitations, we are able to conclude that ECT does not seem to harm cognitive function as assessed by this test battery. On the contrary, we demonstrated cognitive improvement after ECT. The mechanism behind this effect is yet unknown, and future assessments in the current project will elucidate whether the cognitive improvement endures.

## Author Contributions

Both authors designed the study and prepared the manuscript. CM collected the data, performed the statistical analyses, and drafted the paper. Both authors had access to all study data and read and approved the final manuscript.

## Conflict of Interest Statement

The authors declare that the research was conducted in the absence of any commercial or financial relationships that could be construed as a potential conflict of interest.
